# A Design of FPGA-Based Neural Network PID Controller for Motion Control System

**DOI:** 10.3390/s22030889

**Published:** 2022-01-24

**Authors:** Jun Wang, Moudao Li, Weibin Jiang, Yanwei Huang, Ruiquan Lin

**Affiliations:** College of Electrical Engineering and Automation, Fuzhou University, Fuzhou 350116, China; wangjunfzu@fzu.edu.cn (J.W.); N190127108@fzu.edu.cn (M.L.); 210110008@fzu.edu.cn (W.J.); rqlin@fzu.edu.cn (R.L.)

**Keywords:** BPNN, PID, adaptive control, PWM, co-simulation, speed measurement, DC motor, FPGA

## Abstract

In the actual industrial production process, the method of adaptively tuning proportional–integral–derivative (PID) parameters online by neural network can adapt to different characteristics of different controlled objects better than the controller with PID. However, the commonly used microcontroller unit (MCU) cannot meet the application scenarios of real time and high reliability. Therefore, in this paper, a closed-loop motion control system based on BP neural network (BPNN) PID controller by using a Xilinx field programmable gate array (FPGA) solution is proposed. In the design of the controller, it is divided into several sub-modules according to the modular design idea. The forward propagation module is used to complete the forward propagation operation from the input layer to the output layer. The PID module implements the mapping of PID arithmetic to register transfer level (RTL) and is responsible for completing the output of control amount. The main state machine module generates enable signals that control the sequential execution of each sub-module. The error backpropagation and weight update module completes the update of the weights of each layer of the network. The peripheral modules of the control system are divided into two main parts. The speed measurement module completes the acquisition of the output pulse signal of the encoder and the measurement of the motor speed. The pulse width modulation (PWM) signal generation module generates PWM waves with different duty cycles to control the rotation speed of the motor. A co-simulation of Modelsim and Simulink is used to simulate and verify the system, and a test analysis is also performed on the development platform. The results show that the proposed system can realize the self-tuning of PID control parameters, and also has the characteristics of reliable performance, high real-time performance, and strong anti-interference. Compared with MCU, the convergence speed is far more than three orders of magnitude, which proves its superiority.

## 1. Introduction

The PID control algorithm is widely used in practical engineering [1], but when facing the nonlinear and time-varying characteristics of the controlled object, it has the problems of tedious parameter adjustment and poor nonlinear adaptability. Therefore, the limitations of conventional PID in engineering applications are becoming more and more obvious [2,3,4]. The boom in artificial intelligence has led to an increasing focus on neural network control. Neural network has the characteristics of self-learning, self-adaptive and good robustness, etc. Combining PID controller with neural network can meet the actual demand for response speed and stability in the control process. Therefore, it plays an increasingly important role in the field of practical intelligent control [5,6,7,8].

The traditional method of implementing control algorithms in MCU has been suffering from slow convergence and poor real-time performance. In [9], a DC motor speed regulation system based on incremental PID algorithm is proposed with the microcontroller AT89S52 as the control core to achieve stable speed regulation of the DC motor. In [10], the parameters of the fuzzy controller are adjusted using a particle swarm optimization algorithm, and a digital speed control system for DC motors based on the STM32 microcontroller is completed. The results show that the system has the advantages of high reliability and high control accuracy. In [11], a self-driving precision compass based on BP neural network and PID control is designed, and the precision control of the micro DC motor is realized on the STM32F103C8T6 microcontroller. In [12], Using MCU-STM32F1-3 as the main controller, the cascade PID is used to control the flight attitude and can meet the control requirements of take-off, hovering, and landing flight modes. In [9,10,11,12], accurate and stable control of a DC motor is accomplished, but it is deficient for some applications where real-time performance is more demanding.

With the development of integrated circuits and computer technology, the hardware implementation of control algorithms has become possible. In [13], a PID-based motion controller was designed and then implemented on a Basys3 FPGA with higher performance compared to microcontroller and DSP. In [14], a neural network PID controller was designed by Verilog language and applied to the motion control of a service robot to achieve control stability and accuracy. In [15], the neural network sliding mode control algorithm is implemented based on FPGA and a signal monitoring platform is built to collect and display the main control law and PWM control signals. The final experimental results show the feasibility of neural network sliding mode control implemented by FPGA. FPGA can complete multiple operations in one cycle through parallel computing, and the programmability and reconfigurability greatly shorten the design cycle, enabling perfect mapping of neural networks on FPGA [16,17,18,19]. In addition, compared with the microcomputer serial processing method, FPGA has the characteristics of fast speed, low power consumption, and high reliability. Therefore, it is a good idea to implement a neural network PID closed-loop control system based on FPGA.

In view of the most demand for real-time performance and high reliability, this paper proposes, for the first time, a closed-loop motion control system based on a BPNN PID controller by using a Xilinx FPGA solution. The proposed system is structurally divided into two parts: BPNN PID control algorithm design part and the closed-loop control system peripheral module design part. The first part consists of modules such as forward propagation module, PID module, main state machine module, error backpropagation, and weight update module. The forward propagation module consists of several neurons in different layers, which are responsible for completing the forward propagation operations from the input layer to the output layer. The PID module implements the mapping of the incremental PID algorithm to the RTL, which is divided into three sub-modules designed separately according to the structure of the arithmetic, and is responsible for completing the output of the control amount. The main state machine module is responsible for generating enable control signals for each module, and then controlling the operation of each sub-module according to the execution sequence of the algorithm. The error backpropagation and weight update module is designed and implemented with the gradient descent principle as the theoretical basis, which is used to update the weights of each layer of neural network. The second part is divided into two parts: the motor speed measurement module design part and the PWM signal generation module design part. In the design of the motor speed measurement module, the quadruple frequency interface circuit was completed using the frequency doubling technology principle of the encoder, and the actual speed of the motor was measured using the frequency method. The PWM signal generation module is responsible for generating PWM waves with different duty cycles to control the rotation speed of the motor. A co-simulation vertifcation platform of Simulink and Modelsim is built to improve the verification efficiency. The simulation and experiment results show that the designed system can play an effective control of the motor, and has the characteristics of reliable performance, high real-time performance, and strong anti-interference, which shows the validity and the superiority of the proposed system.

## 2. Neural Network PID Controller System

**Figure 1:**
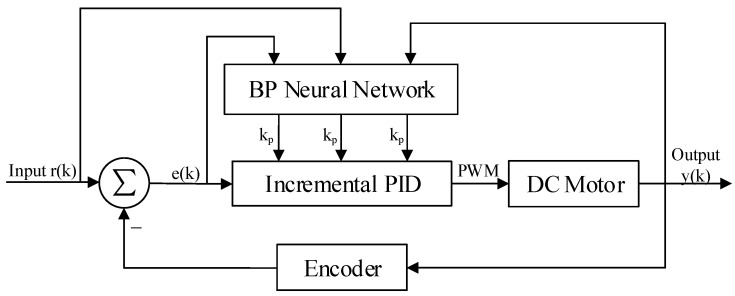
Structure diagram of PID controller system based on BP neural network.

PID control is also known as proportional ($k_{p}$), integral ($k_{i}$), and derivative ($k_{d}$) control [20]. It is widely used in industrial control processes as a mature and effective control algorithm [21]. Its principle is that the system error e(k) between the desired value r(k) and the actual output value y(k) is fed back to the controlled object after the combination of proportional, integral, and differential, so that the actual output value y(k) is constantly close to the desired value r(k), and the tracking control of the controlled object is finally realized. In the actual motion control process, due to the complex control environment and the existence of nonlinear and time-varying characteristics of the controlled object, conventional PID control cannot perform adaptive parameter tuning to achieve good adaptability [22]. Theoretically, BPNN has the dynamic characteristics of self-learning and adaptability, and is not only capable of approximating arbitrary nonlinear function, but also has a simple and clear structure. Therefore, the combination of BPNN and PID control algorithm to achieve the online self-tuning of PID control parameters can achieve the optimal motion control effects. The schematic diagram of the neural network self-tuning PID closed-loop control system is shown in Figure 1.

In Figure 1, the servo motor is the controlled object, while the encoder is responsible for measuring the actual motor speed. The motion state quantity $x_{i}=\left[r\left(k\right),e\left(k\right),y\left(k\right)\right]$ of the system is fed into the BPNN to learn and train, and then an optimal control law is found to finally achieve the actual output value y(k) of the motor to quickly and accurately track the desired value r(k). Among them, the control equation of the incremental digital PID in the adaptive closed-loop control system is shown below:

(1) $$\left\{\begin{array}{c} u(k)=u(k-1)+\Delta u(k)\\ \Delta u\left(k\right)=k_{p}\left[e\left(k\right)-e\left(k-1\right)\right]+k_{i}e\left(k\right)+k_{d}\left[e\left(k\right)-2e\left(k-1\right)+e\left(k-2\right)\right]\\ =k_{p}\Delta e\left(k\right)+k_{i}e\left(k\right)+k_{d}\left[\Delta e\left(k\right)-\Delta e\left(k-1\right)\right] \end{array}\right.$$

The proportional, integral, and derivative of Equation (1) represent the three PID control parameters, while e(k)=r(k)−y(k), e(k−1), and e(k−2) represent the current moment error, upper moment error, and upper upper moment error, respectively. From Equation (1), it can be seen that the incremental digital PID algorithm is only related to the previous three sampling values. Compared with the disadvantages of the positional digital PID, such as large computation and ease of causing integral saturation, this algorithm has the characteristics of low computation and high real-time performance. In the BPNN PID closed-loop control system, $k_{p}$, $k_{i}$, and $k_{d}$ of the incremental PID are adjustable coefficients with the system motion state, so the PID output of the above equation can be described as follows:

(2) $$u\left(k\right)=f[u\left(k-1\right),k_{p},k_{i},k_{d},e\left(k\right),e\left(k-1\right),e\left(k-2\right)]$$

In Equation (2), f(x) is a nonlinear function related to u(k−1), $k_{p}$, $k_{i}$, $k_{d}$, e(k), etc. The controller parameters are adjusted to achieve the optimal performance index by adaptive learning and weighting factor adjustment of the neural network [14].

**Figure 2:**
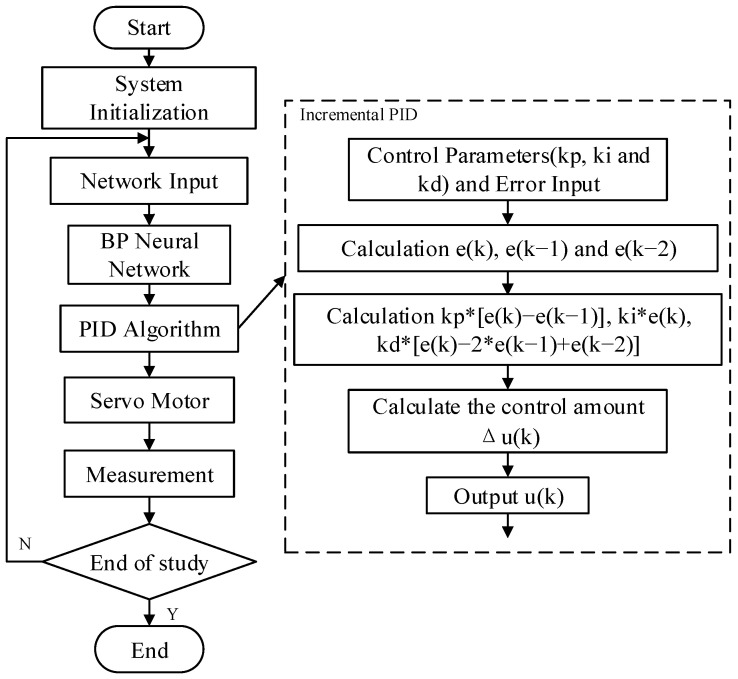
Flow chart of closed-loop control system.

The operation of the entire closed-loop control system is unified and coordinated by the internal clock of FPGA. The system only needs to provide the desired input value r(k), clock, and reset signal to automatically complete the self-tuning process of the PID controller parameters. The flow chart of the system implementation is given in Figure 2. After the system is powered on, the system is first initialized and the motion state quantity is used as the input of BPNN, then the PID control parameters are derived through the forward propagation of the network, and finally the PID controller with the parameters will output the control signal to the controlled object to complete the real-time control of the controlled object. The response effect of the controlled object will be measured by the sensor, and the measurement result will be fed back to the system for the next control calculation. During each cycle, BPNN will learn the control effect of the controlled object by the last output in a targeted manner. This process is the process of error backpropagation to modify the synaptic connection weights until the output response of the system meets the design requirements.

## 3. FPGA Design of BP Neural Network PID Algorithm

**Figure 3:**
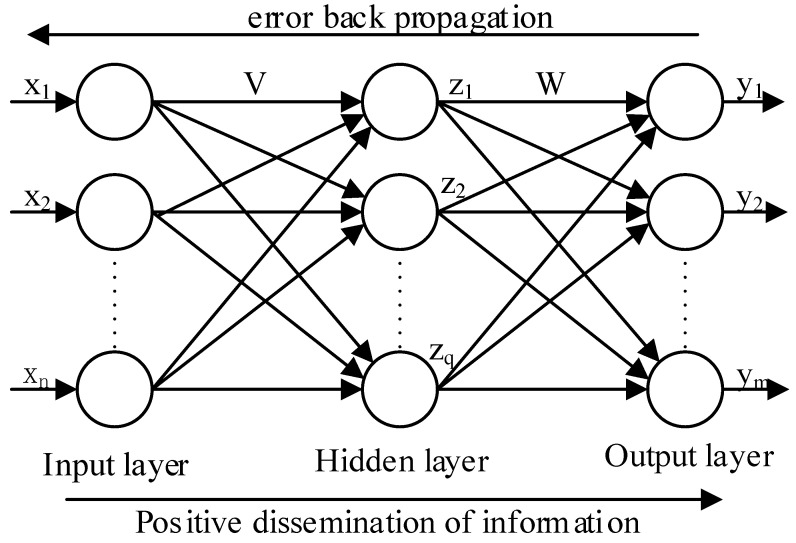
Structure diagram of three-layer BP neural network.

**Figure 4:**
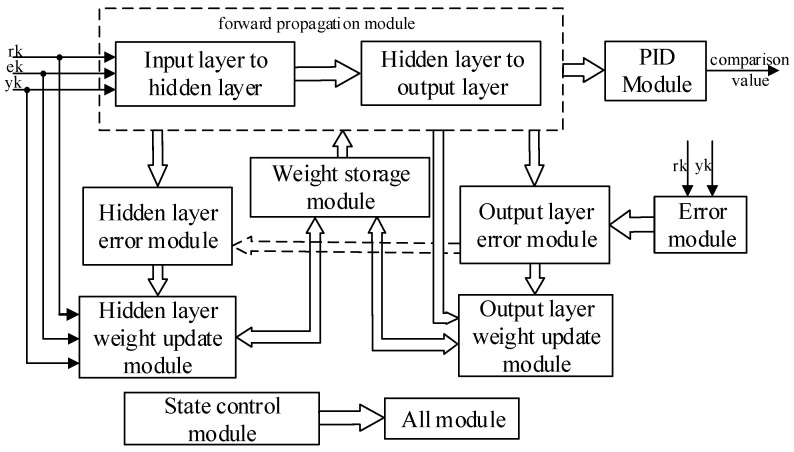
FPGA design framework of BP neural network PID controller.

**Figure 5:**
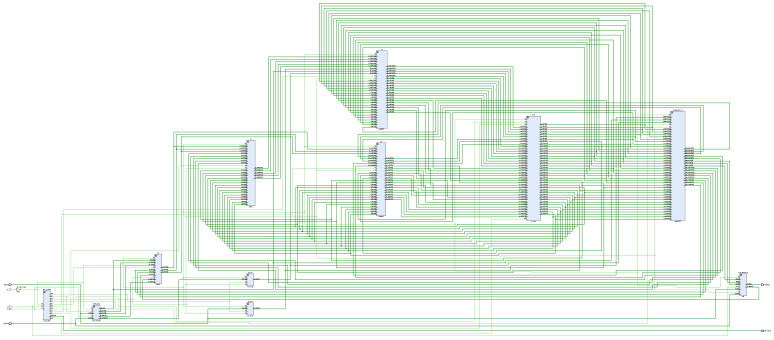
BP neural network PID controller: top-layer RTL schematic.

BPNN consists of input layer, hidden layer, and output layer, which is a multilayer feed-forward neural network. Signal forward propagation and error backpropagation are the main features of BPNN. It modifies the connection weights between each neuron by error backpropagation, and the process of backpropagation is the process of neural network learning [23,24,25,26,27,28]. The topology of the three-layer BPNN is shown in Figure 3, where $x_{i}$ and $y_{j}$ are the input and output of the network, respectively, and $z_{k}$ is the hidden layer output of the network. According to the structure of BPNN, using the idea of top-down design, this paper divides the network into forward propagation module, weight storage module, weight update module, PID module, state control module, error module, etc. The connectivity and training process between the different functional modules is given in Figure 4, and the corresponding RTL view is shown in Figure 5. First, the weights of the motion state quantity and the weight storage module are sent to the BPNN, then the forward calculations of the hidden layer and the output layer neurons, and finally, the PID module outputs the comparison value. The connection weights of each layer of the network are corrected using the gradient descent principle, and the changes of the weights of the corresponding layer are calculated according to the output layer error module and the implied layer error module, respectively. After the weight changes are delivered to the corresponding weight update module to obtain the new weights, the old weights of the weight storage module are replaced with the new weights. The state control module is implemented by a finite state machine, and the enable signal is generated by means of trigger edge triggering. The enable signal controls each sub-module in turn, which can effectively avoid timing conflicts caused by too many modules, and also facilitate timing constraints and timing analysis of the design.

**Figure 6:**
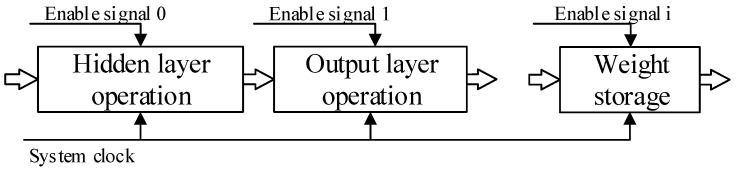
Network execution sequence diagram.

FPGA is used to implement neural networks because its good parallelism is compatible with the way neural networks operate. Generally, it is impossible to implement the neural network structure in parallel in hardware. There must be some sequential execution processes. Therefore, it is necessary to select the most suitable hardware structure to complete the best mapping of the neural network on FPGA [29,30]. As shown in Figure 6, a combination of parallel and serial is proposed in this design to complete the implementation of BPNN on FPGA. Parallel operations are used between the neurons in each layer, and the FPGA pipeline technology is used in the architecture. The pipeline design is mainly a method to reduce the delay of the combinational logic by splitting a larger combinational logic block into multiple small combinational logic blocks and inserting registers between these. An enable signal is also added in each module to start the sequential execution.

### 3.1. Forward Propagation Module Design

**Figure 7:**
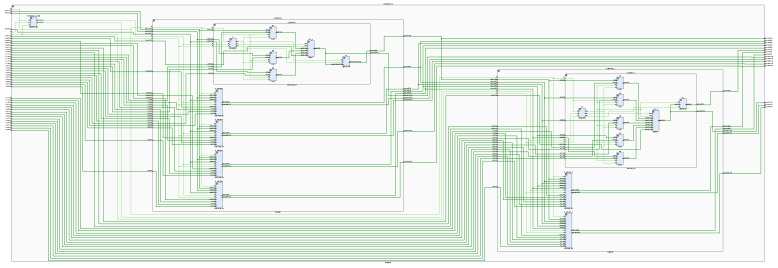
RTL view of the forward propagation module of a BP neural network.

**Figure 8:**
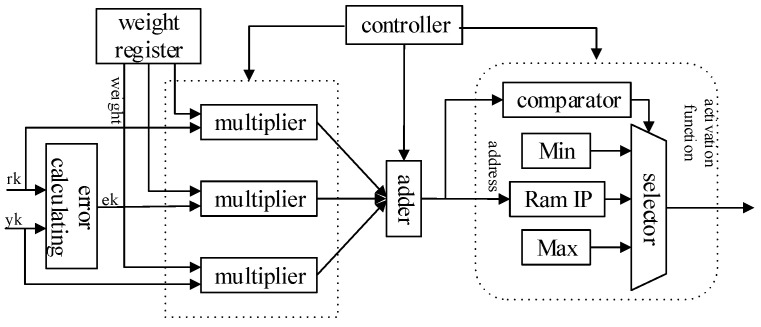
Hardware circuit design of single neuron.

**Figure 9:**
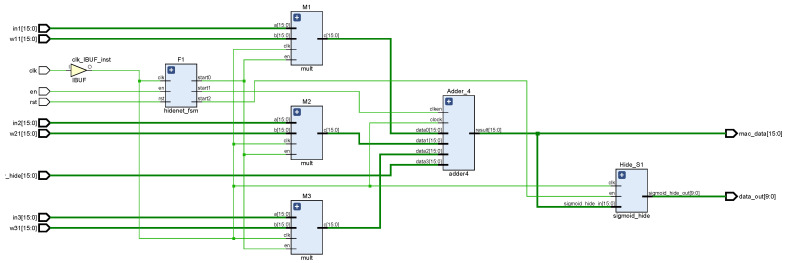
RTL schematic diagram of single neuron.

A neural network is composed of a large number of nodes (or neurons) connected to each other [23]. Therefore, the neuron is the basic unit of the forward propagation module, and its design is crucial to the FPGA realization of the entire network. Figure 7 shows the RTL view of the forward propagation module, including hidden layer operations and output layer operations, and each layer contains several neurons. Figure 8 shows the hidden-layer neuron design of the system. First, the output of the upper-layer neuron is multiplied by the connection weights of the corresponding nodes, then the accumulation process is performed, and finally converted to index address to read the RAM stored value in the activation function module. The stored fixed value is approximated instead of the function value of the activation function, which is used as the input value of the neuron in the lower layer, and then passed sequentially layer by layer until the output layer outputs the control parameters, which indicates the end of forward propagation. The neuron design of the output layer is similar to the neuron design of the hidden layer, but the difference lies in the activation function. Figure 9 shows the schematic of the neuronal RTL after the synthesis of Vivado 2018.3 development software.

In this design, The lookup table (LUT), comparator, and selector are chosen to implement the activation function module. According to the functional properties of the activation function, the interval of the function independent variable is divided into (−∞,−4)∪[−4,4)∪[4,+∞), and the activation function of the hidden layer neuron is shown in the following Equation (3). When the input address is in the range of the interval (−∞,−4)∪[4,+∞), the function value is taken as a fixed value. When the range is in the interval [−4,4), the definition domain is divided into multiple subintervals by using 1/256 as a subinterval length, and then the fixed value corresponding to the input address is approximated to represent the output value of the activation function. The internal operation of the module is a 16-bit fixed-point decimal operation with eight decimal places, while the absolute value of the function value of the activation function of each neuron does not exceed 1. If the function value is stored in RAM as a 16-bit fixed-point decimal, six more bits of storage space will be wasted. Therefore, to save FPGA resources, the function value of the activation function is represented by a 10-bit fixed-point decimal. A COE file with a storage depth of 2048 is generated on MATLAB2019b and used as the initial file of the RAM IP core.

(3) $$h\_tanh\left(x\right)=\left\{\begin{array}{cc} -1 & x<-4\\ \frac{e^{x}-e^{-x}}{e^{x}+e^{-x}} & -4\leq x<4\\ +1 & x\geq 4 \end{array}\right.$$

### 3.2. Error Backpropagation and Weight Update Module Design

**Figure 10:**
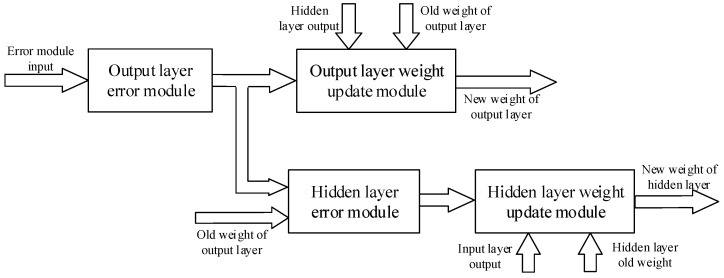
Structure design of backpropagation algorithm.

**Figure 11:**
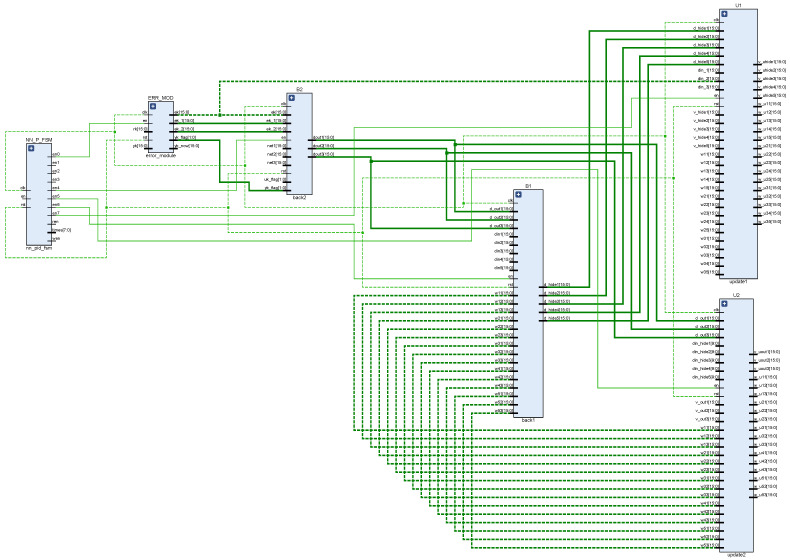
RTL schematic diagram of backpropagation algorithm.

According to the chain rule, the backpropagation algorithm uses the error between the expected and the actual output as the backpropagation, and uses the gradient descent method to adjust the network parameters to promote the error to develop in the direction of smaller [31,32,33]. The principle of the gradient descent algorithm is to find the extreme point of the objective function y=f(x), which is the point where the derivative $f^{\prime }\left(x\right)=0$. In the implementation of the algorithm, the initial value $X_{0}$ is selected, and the value of x is changed by several iterations during the training process, and finally the extreme value point of the function is found after a large number of iterations. The design divides the backpropagation algorithm into four modules, which are the output layer error module, the output layer weight update module, the hidden layer error module, and the hidden layer weight update module. Figure 10 shows the design structure of the backpropagation algorithm, while Figure 11 shows the schematic of this part of the RTL extracted from the top-level RTL view. In the design, the error module compares the actual wheel speed obtained by the encoder with the desired speed to derive the system error, and then the error is back-propagated by the output layer error module, and finally the weight update of the output layer is completed in the output layer weight update module. Based on the node error of the output layer, the node error of the hidden layer is calculated in the hidden layer error module, and then the weight of the hidden layer is updated in the hidden layer weight update module. The network weights updated by the backpropagation algorithm will be stored in the weight storage module for the next network training.

The module is composed of several multipliers and adders. The input values and weights of the network are 16 fixed-point numbers of 8 decimal places. After they are multiplied, they become a 32-bit fixed-point number, and the decimal place becomes 16 bits. In order to abandon unnecessary digits occupying resources, the method of truncation is adopted to keep the output as a 16-bit fixed-point number. In a 16-bit binary number, the highest bit is the sign bit. When it is 0, it means that the value is positive. When it is 1, it means the value is negative.

### 3.3. PID Module Design

**Figure 12:**
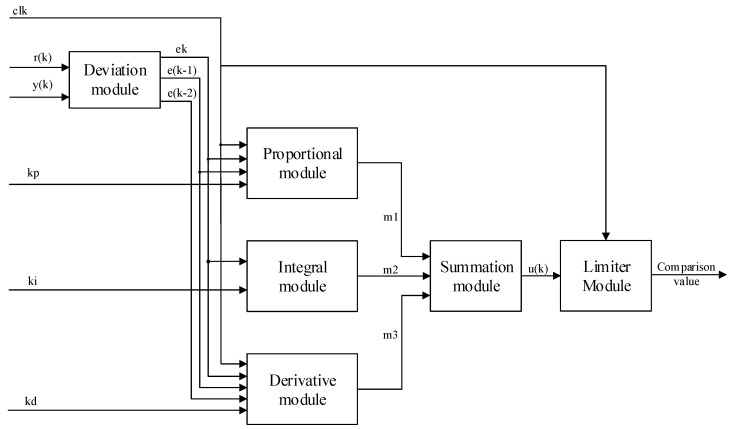
Structure of incremental PID control algorithm.

**Figure 13:**
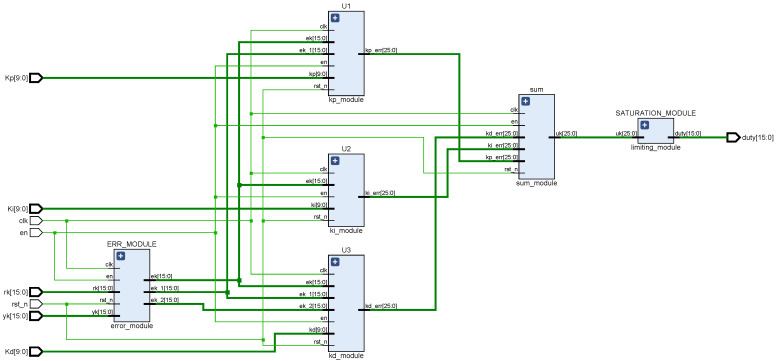
RTL schematic of incremental PID control algorithm.

This design uses FPGA to implement the incremental digital PID algorithm. In the whole design of incremental PID control, only the last three error sampling values e(k), e(k−1), and e(k−2) need to be stored to realize its function [34,35]. According to the mathematical structure of Equation (1), the structure diagram of this incremental PID control algorithm is shown in Figure 12, and the corresponding RTL schematic is shown in Figure 13. In Figure 12, $k_{p}$, $k_{i}$, and $k_{d}$ are the outputs of the network output layer, r(k) and y(k) are the set speed and the current actual speed of the DC motor, respectively, m1, m2, and m3 are the results of the proportional, integral, and derivative parts of the PID equation, respectively, and u(k) represents the output value of the PID control system. The deviation operation is used to obtain the difference e(k), e(k−1), and e(k−2) for a given number of r(k) and y(k), and then the result is fed to the later operation unit for processing. The proportional, integral, and derivative modules multiply $k_{p}$, $k_{i}$, and $k_{d}$ with Δe(k), e(k), and Δe(k)−Δe(k−1), and feed the results to the summation unit for processing. In the summation module, m1, m2, and m3 are summed to obtain the control increment Δu(k) of the PID, and then the value of u(k−1) stored in the register is added to finally obtain the value of u(k) of the PID control arithmetic. The limiter module consists of a comparator to limit the comparison values delivered to the PWM module to saturate the output with the set maximum value when it reaches its maximum value.

The PID control module has a bit width of 16 bits for both r(k) and y(k), and a bit width of 10 bits for the other three parameters $k_{p}$, $k_{i}$, and $k_{d}$. In order to discard the space occupied by unnecessary bits, when using FPGA to implement the multiplication and addition operations in the PID control algorithm for fixed-point calculations, an output conversion part was added to the program so that the value of the output result is also 16-bit bit wide. The format of fixed-point decimal is that the highest bit indicates the sign bit, and the remaining bits indicate the value bit, where the decimal bit is 8 bits. When the highest bit is 0, it is positive, and when the highest bit is 1, it is negative.

### 3.4. Main State Machine Module Design

**Figure 14:**
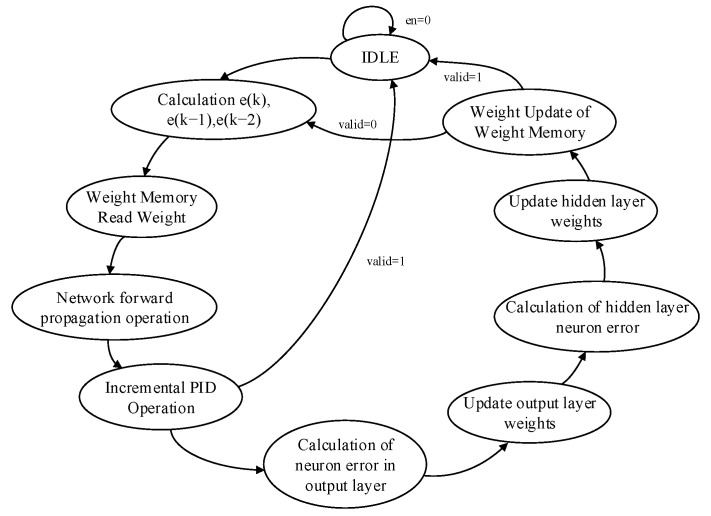
The main state machine diagram of the BP neural network.

The structure used in this design BPNN is a three-layer network structure, which contains three neurons in the input layer, five neurons in the hidden layer, and three neurons in the output layer corresponding to the proportional, integral, and differential control coefficients of the PID. In the process of complex circuit design, the design of state machines is one of the essential parts [36] of the conversion process from mathematical algorithm to RTL design. The main state machine generates the enable signals for different modules by jumping through the states, which are then transferred to the next processing module. The main state machine of this adaptive closed-loop motion control system is shown in Figure 14, and the process of implementing the main parts of BPNN PID control is described as follows.

The actual output value y(k) of the DC motor is first sampled, then the errors at moments *k*, k−1, and k−2 are calculated, then the connection weights of each layer of the network are read from the weights register, then the forward propagation operation from the input layer to the output layer is completed, and finally the three control parameters are output. The updated control parameters are used to obtain the PWM wave control signal by PID operation, and the motion control of the DC motor is achieved by the drive control board. The backpropagation is based on the gradient descent principle to correct the weights of each layer of the network and update the weights layer by layer from the output layer to the input layer in turn. According to its principle, the error of the corresponding layer is obtained, and then the connection weight of the corresponding layer is updated. After the weight of each layer of the network is updated, the training process ends. After completing a training session, let k=k+1 at this point, and then start the next learning training until the required number of training sessions is reached or the error meets the requirement, then the training is finished.

## 4. Design of Peripheral Modules for Neural Network PID Closed-Loop Control System

**Figure 15:**
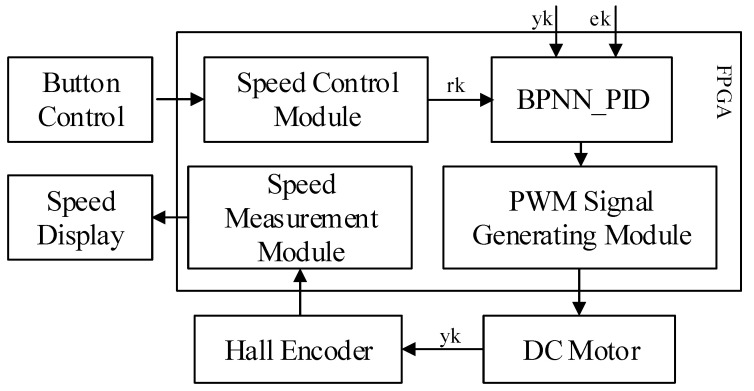
DC motor control system measurement and control module structure diagram.

In this proposed system, the neural network PID controller is used to realize the closed-loop motion control of the DC motor, and the proposed peripheral module consists of speed control module, PWM signal generation module, speed measurement module, etc. The module structure is shown in Figure 15. Due to the mechanical characteristics, there is jitter when the key is pressed or released, and key dejittering is accomplished by counting to a predetermined value (15 ms) by a counter. The speed control module detects the key value to complete the motor speed setting and uses the desired value r(k) as an input value of the neural network. The BPNN_PID module is the BPNN PID controller designed above, with output comparison values in the range of 0–4999, where y(k) is the actual output value of the motor and e(k) is the deviation value of the system. The PWM signal generation module generates PWM pulses with different duty cycle according to the comparison value, and the pulses are input of the motor driver board to complete the operation control of the DC motor. The speed measurement module is responsible for measuring the real-time speed of the DC motor by sampling the total number of rising and falling edges of the A and B phase quadrature pulse signals over a period of time $T_{c}$, and then calculating them by the *M* speed measurement method (frequency method) to complete the measurement of the real-time speed of the DC motor.

### 4.1. Motor Speed Measurement Module Design

**Figure 16:**
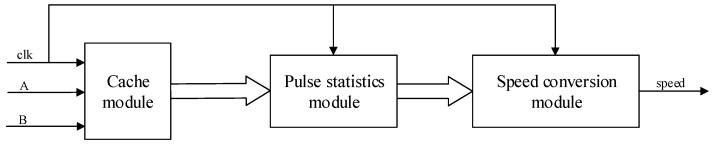
Design structure diagram of DC motor speed measurement module.

**Figure 17:**
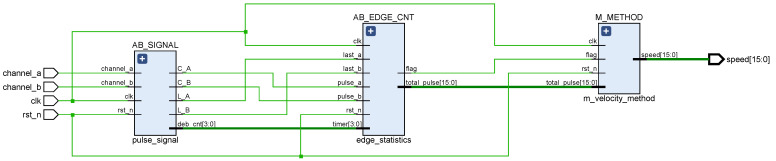
Speed measurement module RTL schematic.

According to the principle of DC motor speed measurement, we can design the speed measurement module by referring to the design method of *M* speed measurement method and displaying the result of speed measurement by digital tube. For a deterministic encoder, the A and B phase pulse signals jump twice in one cycle T, and the four jumps are evenly distributed in phase. Therefore, the encoder measurement accuracy can be improved by generating quadruple frequency signals with four hops and counting them. The key to the design of the speed measurement module is to be able to accurately capture the rising and falling edges of the A and B signals and to complete the count of the number of pulses. Figure 16 shows the structure of the speed measurement module, which is divided into cache module, pulse statistics module, and speed conversion module. Figure 17 is the RTL schematic diagram of the speed measurement module, corresponding to the three sub-modules of the design structure diagram. In the schematic, the AB_SIGNAL module is responsible for sampling the A and B signals, the AB_EDGE_CNT module is responsible for counting the total number of rising and falling edges of the A and B signals within Tc = 10 ms, and the M_METHOD module is responsible for converting the total number of pulses into motor speed.

This rst_n is the system low reset signal, clk is the system clock divided clock signal (frequency F = 50 M), and channel_a and channel_b are the A and B signals, respectively. Considering that there may be jitter and burr in the A and B signal when the level jumps, the C_A and C_B registers, respectively, store the level value of the tenth system clock after the A and B signal level reversal, that is, when deb_cnt = 10, while L_A and L_B register the level value before the A and B signal level reversal. In addition, deb_cnt counts up to 10, and when the A and B phase pulse signal levels are flipped, deb_cnt clears and starts counting again. In the AB_EDGE_CNT module, when deb_cnt = 10 and C_A⊕L_B = 1, the edge statistics value is added by 1. Conversely, when deb_cnt = 10 and C_B⊕L_A = 1, the edge statistics value is subtracted by 1. The total_pulse is the total number of level flips of A and B signals during the sampling time period. When the counter count reaches F∗Tc, the flag flag is set to 1 and total_pulse is cleared to zero. The M_METHOD module is based on the statistical value of total_pulse, then based on the known number of fixed pulses generated by one rotation of the DC motor, with a known sampling time Tc, and finally by the calculation of the frequency method, the current speed of the motor speed (15:0).

### 4.2. PWM Signal Generation Module Design

**Figure 18:**
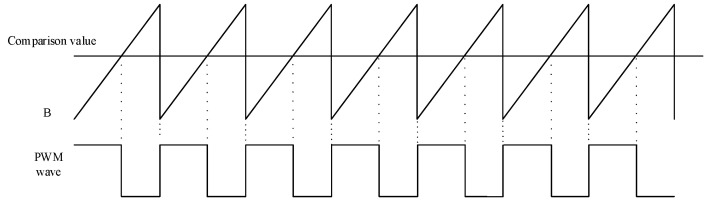
PWM signal waveform generation.

The pulse-modulated PWM signal is generally generated by an analog comparator. A given reference voltage is connected to one end of the comparator and a periodic sawtooth wave voltage is connected to the other end. When the sawtooth voltage is less than the reference voltage, the output level is high, and when the ramp voltage is greater than the reference voltage, the output level is low, so that the duty cycle of the PWM signal can be changed by changing the reference voltage. Based on this idea, the PWM waveform is generated by FPGA, and only internal FPGA resources are needed to replace the analog comparator with a digital comparator, eliminating the need for an external D/A converter and analog comparator compared to an analog controller. The PWM signal is generated as shown in Figure 18. The sawtooth wave signal B, when compared with the fixed value A, is able to generate a PWM signal with a fixed pulse width. By changing the value of A, we can change the duty cycle of the PWM signal. When the set speed value of the motor changes, the duty cycle of the PWM signal changes as well. When the duty cycle of the PWM signal increases, the motor speed speeds up, and when the duty cycle of the PWM signal decreases, the motor speed slows down.

**Figure 19:**
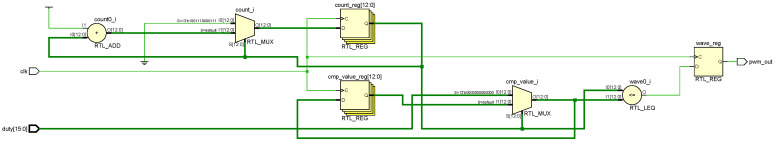
RTL schematic diagram of PWM signal generation module.

Figure 19 shows the RTL schematic of the PWM signal generation module. In the design of the PWM waveform, the counter outputs counting pulses under the excitation of the clock signal CLK. In order to output a gradually increasing sawtooth waveform, the program outputs a count value at the arrival of each rising edge of the clock and adds 1 at the arrival of the next rising edge of the clock until the count is cleared to zero when count = “1001110000111”, thus, a periodic sawtooth waveform is output. The output signal count of the sawtooth wave and the comparison value duty of the BPNN_PID output are added to both input ports of the digital comparator at the same time, and then the two are compared. If the value of count is less than the value of duty, the comparator outputs high, and, vice versa, it outputs low. This can generate a periodic PWM signal, and as long as the comparison value duty of BPNN_PID output is changed, the duty cycle of PWM signal can be changed to achieve the purpose of speed regulation.

## 5. Simulation and Implementation of Closed-Loop Control System

### 5.1. Co-Simulation of Closed-Loop Control Systems

**Figure 20:**
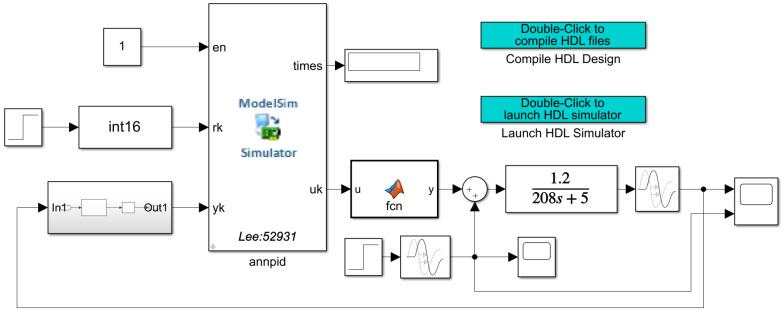
Simulink simulation model of closed loop control system.

**Figure 21:**
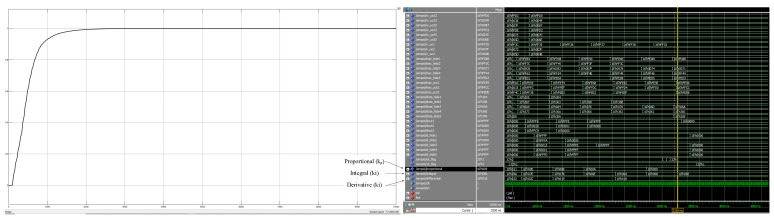
Step response curve graph and control parameter change graph without interference.

**Figure 22:**
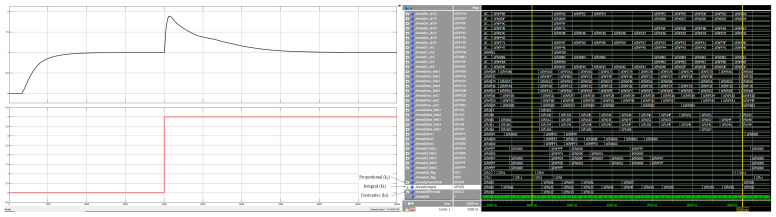
Step response curve and control parameter change diagram after adding interference at *t* = 4 μs.

To address the problem of real-time simulation of a closed-loop motion control system, this paper adopts a co-simulation method combining Simulink and Modelsim to verify the correctness and effectiveness of the adaptive closed-loop control system design in this paper. IP core is used in the design, so a co-simulation environment needs to be configured. First, we use Modelsim to compile the corresponding IP source files to generate simulation library files, then add the generated files to the project, and finally create the model to be simulated in the Simulink platform. Closed-loop control systems are internally calculated with 16 fixed-point fractional bits, where the highest bit is the sign bit, the lower eight bits are the fractional bits, and the remaining seven bits are integer bits. The transfer function selected for the closed-loop control system is shown below. The Simulink simulation model created by using Matlab 2019b is shown in Figure 20, and the unit step response curves and control parameter variation results of its closed-loop control system are shown in Figure 21. In order to test the immunity of the adaptive closed-loop control system designed here, the simulation curve change results are shown in Figure 22 after adding the disturbance source. The simulation frequency of the whole system is set to 50 MHZ, and the simulation duration of Simulink is set to 10,000 ns.

(4) $$G\left(s\right)=\frac{Y(s)}{U(s)}=\frac{1.2}{208s+5}e^{-10s}$$

As can be seen from the step response graph (left panel) of Figure 21, the actual output value continuously converges to the target value during the adaptive regulation of the neural network, and finally the output reaches the desired input value of 1 at around 3000 ns. In addition, the Modelsim simulation waveform in Figure 21 (right panel) shows that the PID control parameters (proportional, integral, and derivative) remain essentially constant after 3000 ns. At this point, $k_{p}=9/256$, $k_{i}=102/256$, and $k_{d}=24/256$. By adding a disturbance signal with a constant of 4 at *t* = 4 μs (bottom left), it can be seen from the Modelsim simulation plot of Figure 22 (right) that after 5.25 μs, the PID control parameters regain stability after retraining the output of the network and the actual output of the system (top left) is able to track the input signal again. At this point, $k_{p}=6/256$, $k_{i}=82/256$, and $k_{d}=18/256$. This proves the correctness and effectiveness of the neural network PID controller design. Meanwhile, the FPGA-based neural network PID controller also has the advantages of self-tuning parameters, as well as fast regulation times and small steady-state errors, and is highly resistant to interference. References [9,10,11] use a microcomputer as the experimental platform to design and implement several different control algorithms for DC motor control systems, and achieve stable and reliable control results. However, the control times are in excess of several hundred milliseconds, whereas the control system designed in this paper is able to reduce the control time to a few microseconds, so the proposed design is superior and provides a reference for complex practical control environments.

### 5.2. FPGA Implementation of Closed-Loop Control System

**Figure 23:**
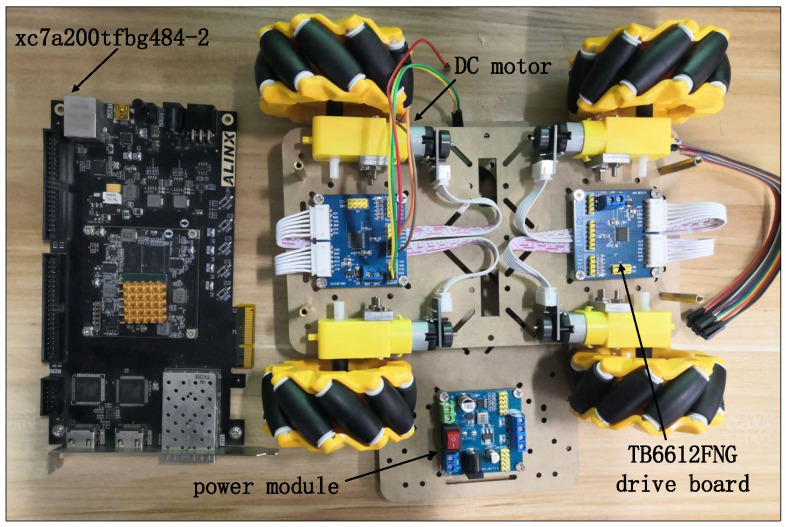
Experimental development platform for FPGA-based closed-loop control system.

**Figure 24:**
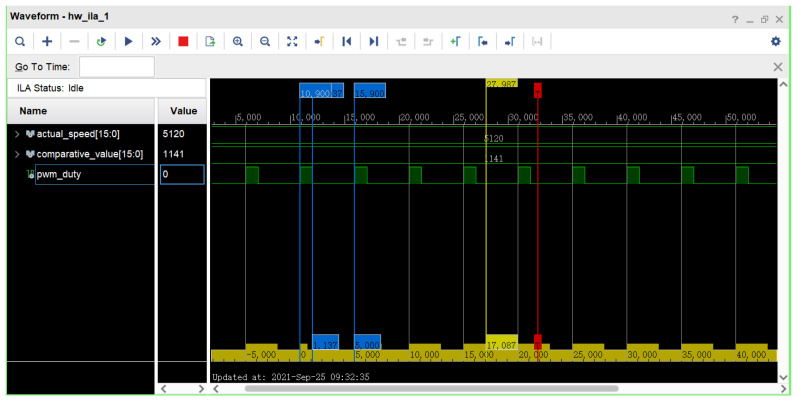
Embedded logic analyzer (ILA) acquisition signal diagram.

The experimental object of this proposed system is a DC motor with encoder, which generates 120 × 13 = 1560 fixed pulses per revolution, while the motor encoder outputs quadrature pulse signals of A and B phases. The hardware development platform is the Xilinx model Xc7a200tfbg484-2 FPGA development platform. In addition, the operating frequency of the entire control system is configured as the clock frequency F = 50 M. The driver board of the motor is the TB6612FNG DC motor driver board, which is able to control two DC motors at the same time. The A and B phase pulse signals from the encoder are sampled on the FPGA development platform to complete the speed measurement of the DC motor. This proposed system uses an integrated logic analyzer (ILA) to test and analyze the system. Figure 23 shows the development experiment platform of this system. The bit file is first generated in Vivado 2018.3 development software and downloaded to the FPGA development board, and then the signals to be observed are added to the integrated logic analyzer (ILA). Figure 24 shows the actual speed signal, the comparison value signal, and the PWM wave signal collected by the integrated logic analyzer.

In the experimental platform, the given speed of the DC motor is 20 r/min. From the values of the signals shown in Figure 24, it can be seen that the actual speed value (actual_speed (15:0)) of the DC motor collected by the ILA is 5120, and the internal operations of the FPGA are 16-bit fixed-point decimal, with 8 decimal places, so the current DC motor can be obtained by mathematical conversion. The actual speed is 5120/256 = 20 r/min. The period of PWM is set to 10 KHZ, and the high level of PWM wave (pwm_duty) is measured to be 1137 ns, so the duty cycle of PWM wave at the current speed of DC motor can be calculated to be 1137/5000 = 22.74%. From the experimental results, it can be seen that the experimental results verify the correctness and feasibility of the motion control system design. Therefore, the combination of the BPNN and PID control algorithm can achieve the purpose of adaptive closed-loop control, which provides a reference for intelligent motion control.

## 6. Conclusions

The commonly used MCU, such as C8051 or STM32, cannot meet the high requirements for real-time performance and reliability. An innovative neural network PID control method based on FPGA implementation is proposed, and a complete closed-loop control system is implemented. For the design of the BPNN PID controller, a top-down design is used to divide the algorithm into several sub-modules, and the functions and design flow of each module are described in detail. The peripheral module is designed to realize the acquisition of the encoder output pulse signal and the measurement and control of the motor speed. The co-simulation combining Modelsim and Simulink is used to improve the efficiency of verification, and the test verification is completed on the experimental platform. The results show that the designed system can realize the self-tuning of PID control parameters, and also has the characteristics of reliable performance, good control effect, and good robustness. Compared to traditional MCU-based control methods, the convergence speed of the FPGA-based adaptive control method is much faster than three orders of magnitude, proving its superiority over traditional methods.

In this paper, although the design of the adaptive closed-loop motion control system is completed, as well as the system construction and simulation, which confirms its feasibility and reasonableness, there is still a need for continuous exploration and improvement in FPGA resource utilization and human–computer interaction, such as the use of more accurate and less resource-consuming activation function implementation methods, and the possibility of designing remote wireless touch screens to improve operability.

## Data Availability

Not applicable.
